# Detection of placenta accreta spectrum and prediction of adverse perinatal outcomes in pregnant women with placenta previa using ultrasonography and magnetic resonance imaging: A retrospective cohort study

**DOI:** 10.1371/journal.pone.0349503

**Published:** 2026-05-29

**Authors:** Suhra Kim, Yun Ji Jung, Hyun-Soo Zhang, Ju-hee Yoon, Seowon Choi, Hayan Kwon, Ja-Young Kwon, Young-Han Kim, JoonHo Lee

**Affiliations:** 1 Department of Obstetrics and Gynecology, Institute of Women’s Life Medical Science, Yonsei University College of Medicine‌‌, Seoul, Republic of Korea; 2 Biostatistics Collaboration Unit, Department of Biomedical Systems Informatics, Yonsei University College of Medicine, Seoul, Republic of Korea; Xiangya Hospital Central South University, CHINA

## Abstract

**Background:**

Placenta accreta spectrum (PAS), an abnormal placental invasion into the myometrium or beyond the uterine serosa, is associated with adverse pregnancy outcomes. Placenta previa is the most significant individual risk factor for PAS, and Ultrasonography (US) and Magnetic resonance imaging (MRI) are widely used to detecting PAS. However, limited data exist on the effectiveness of US and MRI in predicting maternal and neonatal morbidity. This study evaluated the utility of these imaging modalities, not only in detecting PAS but also in predicting adverse perinatal outcomes.

**Methods:**

This retrospective cohort study included 150 pregnant women with placenta previa who underwent US and MRI at a tertiary hospital between December 2019 and December 2023. PAS was diagnosed clinically or histopathologically after delivery. The predictive performance of US, MRI, and their combination was assessed using McNemar’s test, receiver operating characteristic analysis, and trend analysis.

**Results:**

Forty-one patients were diagnosed with PAS. The PAS group had significantly higher rates of prior caesarean section, maternal hemorrhagic outcomes, and neonatal complications than the non-PAS group. PAS-suspected on US group also showed increased estimated blood loss (EBL) and number of transfusion units administered, as well as a higher incidence of transfusion requirement, use of intrauterine balloon tamponade, hysterectomy, preterm birth before 37 weeks of gestation, neonatal ventilatory support and 1-minutes Apgar scores (AS) <7 compared to those with PAS-unsuspected on US group. PAS-suspected on MRI group had a significantly increased number of transfusion units administered and higher rates of hysterectomy, maternal ICU admission, preterm birth and neonatal complications. US plus MRI yielded progressively higher detection rates, outperforming either modality alone. A linear trend was observed in the increase of adverse perinatal outcomes when PAS was suspected on both US and MRI.

**Conclusions:**

US and MRI showed complementary strengths. US, but not MRI, is informative for predicting higher EBL, transfusion requirement, and use of intrauterine balloon tamponade, whereas MRI, but not US, predicts maternal ICU admission, preterm birth before 34 weeks of gestation, NICU admission, and 5-minute AS <7. Combining both imaging modalities yields better performance than either modality alone in detecting PAS and predicting adverse perinatal outcomes.

## Introduction

Placenta accreta spectrum (PAS) refers to a condition characterized by abnormal placental adherence to the myometrium or invasion into surrounding structures. This abnormal implantation impedes normal detachment of the placenta after delivery, often leading to life-threatening obstetric complications, such as massive hemorrhage, uterine rupture, and the need for hysterectomy [1]. PAS is classified into three types based on the extent of villi invasion: placenta accreta, where the villi attach to the myometrial surface without penetration; placenta increta, involving villi invasion into the myometrium; and placenta percreta, where the villi penetrate the uterine serosa and may involve adjacent organs [2]. Disruptions in the uterine cavity can damage the endometrial–myometrial interface, promoting scar tissue formation and elevating the risk of PAS [3,4].

Placenta previa is the most significant individual risk factor for PAS, with an 11.0-fold increased odds ratio compared to cases without previa [4]. This risk is pronounced when the placenta overlies a uterine scar. Although prior caesarean section independently contributes to the risk of PAS, the coexistence of placenta previa and a history of caesarean delivery leads to a synergistic effect, dramatically elevating the likelihood of PAS [4–6]. The incidence of PAS has increased 10-fold over the past four decades [6].

Accurate prenatal detection of PAS is essential because it affects maternal morbidity and mortality and is associated with a high risk of prolonged surgeries, uncontrollable bleeding, extended hospitalizations, intensive care unit (ICU) admissions, and even death. Patients with suspected PAS should receive comprehensive prenatal counselling, with particular emphasis on the potential need for hysterectomy, which causes permanent loss of fertility. Additionally, clinical outcomes improve considerably when delivery is strategically planned and managed by a specialized multidisciplinary team, preferably at centers of excellence for PAS [7–10].

Detection of PAS relies on imaging techniques, and ultrasonography (US) is a critical imaging modality for its diagnosis [11,12]. As a non-invasive technique, US can be performed repeatedly during pregnancy, facilitating continuous monitoring and timely identification of placental invasion. Although US is a reliable method utilized to detect PAS, its diagnostic accuracy may be compromised under specific circumstances. The effectiveness of US depends on the proficiency of the operator in conducting examinations and interpreting the results. Visualization of the placenta may also be challenging in cases of maternal obesity, posterior placental location, or when placental invasion or adherence is limited or focal [13–15].

Magnetic resonance imaging (MRI) may be considered a complementary imaging modality for assessing the depth of placental invasion and extent of lateral extension, while also enhancing visualization in cases of a posteriorly located placenta [16,17]. The diagnostic value of US and MRI has been reported in the detection of PAS [14,18–23]. Fratelli et al. and D’antonio et al. assert that US and MRI demonstrate similar diagnostic accuracies for PAS detection [21,24]. However, owing to the high cost of MRI, it was performed only in cases where PAS was strongly suspected based on US findings.

US and MRI are, therefore, widely used tools for diagnosing and detecting PAS; however, a lack of data exists on the effectiveness of US and MRI in predicting maternal and neonatal morbidity. While a single study has demonstrated the utility of these imaging modalities in forecasting adverse perinatal outcomes [25], another has reported limited prognostic value [26]. This study aimed to determine whether the combined use of US and MRI improves the prediction of adverse perinatal outcomes, in addition to enhancing diagnostic accuracy for PAS.

## Materials and methods

### Study design and procedure‌‌

A retrospective cohort study was conducted on 150 patients referred to the Department of Obstetrics and Gynecology at our tertiary center for placenta previa, who underwent MRI and delivered at our hospital between December 2019 and December 2023. Data were gathered from electronic medical records, including prenatal US and MRI findings, clinical and pathological observations, maternal complications, and neonatal outcomes. This study was approved by the Institutional Review Board of Yonsei University Health System (approval No. 4-2024-1464, approved on 13 January 2025) and was performed in accordance with the principles of the Declaration of Helsinki. Data were accessed for research purposes between 14/01/2025 and 31/08/2025. The requirement for informed consent was waived by the Institutional Review Board due to the retrospective nature of the study. The authors did not have access to information that could identify individual participants during or after data collection, as all data were anonymized prior to analysis.

### Definitive diagnosis

A definitive diagnosis of PAS was established either clinically or through subsequent histopathological analysis. According to the International Federation of Gynecology and Obstetrics guidelines, PAS was identified when attempts at manual placental separation provoked excessive hemorrhage from the implantation site that was refractory to medical management and required mechanical or surgical intervention [27]. Histopathological confirmation was based on examination of hysterectomy specimens revealing the absence of the decidua basalis and direct trophoblastic invasion into or through the myometrium [27]. All cases were classified according to the FIGO classification system [27].

### US acquisition protocol and analysis

All US placental examinations were performed by maternal–fetal medicine (MFM) specialists, MFM fellows, and expert sonographers with > 10 years of advanced scanning experience in prenatal diagnosis. US examinations were performed using a multiplanar approach with 2–7 MHz transabdominal probes, and transvaginal US was performed using a 5–10 MHz probe with two-dimensional greyscale and color Doppler imaging. Examinations were performed using the WS80A or HERA W10 (Samsung Medison, Seoul, Republic of Korea) and Voluson E10 (GE Healthcare, Milwaukee, WI, USA) US systems.

Placenta previa was classified into the following categories based on the position of the placenta in relation to the internal cervical os: (1) low-lying, (2) marginal, (3) partial, and (4) complete [28]. US indicators of suspected PAS were assessed based on the criteria established by Shainker et al. [29] and Collins et al. [30], including (1) lacunae, (2) loss of a clear zone, (3) uteroplacental hypervascularity, (4) myometrial thinning, and (5) uterovesicular hypervascularity. The presence of at least two US indicator (US ≥ 2) was considered suspicious for PAS. Three obstetricians with specialized training in placental US independently evaluated the presence of PAS without access to the clinical information or histopathological backgrounds.

### MRI acquisition protocol and analysis

We included 150 gravid patients who underwent 1.5 T unit MRI (Achieva dStream 1.5T, Philips Healthcare, Best, the Netherlands) examinations lasting less than 30 minutes without the use of intravenous contrast agents. The following MR sequences were acquired: a single-shot turbo-spin-echo T2-weighted sequence (field of view [FOV], 450 × 500 mm; matrix: 300 × 200; slice thickness, 3 mm; number of slices, 100; flip angle: 90°; gap, 0; repetition time/echo time [TR/TE] = 456/160 ms) in the sagittal plane, a T2-weighted sequence (FOV 400 × 320 mm; matrix: 300 × 240; slice thickness, 4 mm; number of slices, 110; flip angle: 90°; gap, 0; TR/TE = 499/160 ms) in the axial plane, and a T2-weighted sequence (FOV 500 × 500 mm; matrix: 320 × 200; slice thickness, 3 mm; number of slices, 70; flip angle: 90°; gap, 0; TR/TE = 509/160 ms) in the coronal plane. A spectral attenuated inversion recovery T1-weighted sequence was also performed as follows: FOV, 400 × 320 mm; matrix, 260 × 170; slice thickness, 6 mm; number of slices, 60; flip angle, 90°; gap, 0; TR/TE, 666/12 ms.

The acquired magnetic resonance (MR) images were interpreted by two experienced radiologists specializing in reviewing genitourinary MR images without knowledge of the clinical history, pathological diagnosis, and US findings. The MRI assessments were extracted from clinical radiology reports, and the reports were originally generated based on a consensus interpretation by two board-certified radiologists. Radiologists systematically documented factors suggestive of PAS, as described in previous reports [31–33], including the following: (1) dark intraplacental band, (2) lumpy placental borders, (3) intraplacental vascularity, (4) myometrial thinning, (5) extrauterine invasion, (6) placental heterogeneity, (7) uterine bulging, (8) hypervascularity of the uterine serosa and parametrium, (9) bladder tenting, and (10) myometrium disruption. MRI findings were comprehensively and qualitatively assessed in cases with one or more imaging features suggestive of PAS, based on an integrated evaluation rather than a simple count of individual MRI signs. In case of disagreement between the two radiologists regarding the presence of PAS, a final MRI diagnosis was established by consensus.

### Statistical analysis

Continuous variables were summarized as means with standard deviation, and categorical variables were reported as numbers and percentages. Variable comparisons by PAS status and distributions of maternal or neonatal outcomes were conducted using the *t*-test or Wilcoxon rank-sum test for continuous variables and the chi-square test or Fisher’s exact test for categorical variables, as appropriate.

To assess PAS diagnostic performance, sensitivity, specificity, positive predictive value (PPV), negative predictive value (NPV), and overall accuracy were calculated from 2-by-2 contingency tables for US and MRI. McNemar’s test of paired proportion differences was conducted because all patients underwent both US and MRI examinations. Two additional McNemar’s tests using the final PAS status confirmed after delivery (Yes/No) were conducted to assess the differences in sensitivity and specificity between US and MRI.

According to the relative sensitivity and specificity of US and MRI, a combined variable “US + MRI” was created as follows: 0 = both imaging techniques detect PAS negative; 1 = US detects positive, but MRI detects negative; 2 = MRI detects positive, but US detects negative; 3 = both techniques detect positive. Univariate logistic regression models, including US alone, MRI alone, or US + MRI, were employed to evaluate PAS detection. DeLong’s test was used to determine statistically significantly different areas under the receiver operating characteristic (ROC) curve (AUC) [34]. The three corresponding ROC curves were overlaid to visually compare the predictive performances of US alone, MRI alone, and US + MRI.

The trend in PAS prevalence was evaluated across a three-level ordinal risk scale: 0 (US = 0, MRI = 0), 1 (US = 1, MRI = 0 or US = 0, MRI = 1), and 2 (US = 1, MRI = 1). Discordant cases (risk identified on only one imaging modality) were consolidated into the intermediate category (risk scale 1, *n* = 63) to ensure adequate cell counts and preserve the asymptotic validity of the linear-by-linear association chi-square test. For continuous variables, trends across the same ordinal risk scale were assessed using the Jonckheere–Terpstra test, given the non-normal distribution of these variables.

All statistical tests were two-sided, with a *p*-value < 0.05 considered statistically significant. Analyses were conducted using R version 4.4.3 (R Foundation for Statistical Computing, Vienna, Austria).

## Results

In total, 150 patients with placenta previa who underwent placenta MRI were identified at our tertiary hospital. Of the 150 patients, forty-one patients were diagnosed with PAS clinically and histopathologically according to the FIGO classification. Among the histopathologically PAS-confirmed cases (*n* = 13), two were diagnosed as placenta increta, and no cases of placenta percreta were identified. Table 1 summarizes the demographic and clinical characteristics of the patients. There were significant differences between PAS-diagnosed group and non-PAS group in multiple clinical parameters, including previa type (*p* = 0.017), history of previous caesarean section (39.0% vs 14.7%, *p* = 0.003), estimated blood loss (EBL) (1627.8 ± 962.8 mL vs 958.3 ± 528.9 mL, *p* < 0.001), pre- to post-operative hemoglobin (Hb) difference (3.0 ± 1.4 g/dL vs 2.4 ± 1.2 g/dL, *p* = 0.009), transfusion requirement (56.0% vs 37.6%, *p* = 0.041), number of transfusion units administered (2.5 ± 3.5 vs 0.9 ± 1.6, *p* = 0.008), incidence of hysterectomy (31.7% vs 0.9%, *p* < 0.001), maternal ICU admission (12.2% vs 0.9%, *p* = 0.007), preterm birth (gestational age [GA] < 37 weeks) (51.2% vs 27.5%, *p* = 0.006), low birthweight (< 2,500 g) (29.3% vs 13.8%, *p* = 0.028), 1-minute Apgar scores (AS) < 7 (75.6% vs 55.0%, *p* = 0.022), and 5-minute AS < 7 (22.0% vs 3.7%, *p* = 0.001).

**Table 1:** Clinical characteristics and pregnancy outcomes according to PAS status.

Descriptive statistics	Non-PAS (*n* = 109)	PAS (*n* = 41)	*p*-value
**Clinical characteristics**			
Age (years)	36.1 ± 3.8	35.9 ± 4.0	0.757
GA at delivery (weeks)	36.8 ± 1.2	36.2 ± 1.5	0.049
GA on MRI (weeks)	31.1 ± 2.7	30.1 ± 3.0	0.050
Previa (type)			0.017
Complete	60 (55.0)	32 (78.0)	
Partial	11 (10.1)	5 (12.2)	
Marginal	23 (21.1)	1 (2.4)	
Low-lying	15 (13.8)	3 (7.3)	
Previous CS	16 (14.7)	16 (39.0)	0.003
Number of CS			0.001
0	93 (85.3)	25 (61.0)	
1	16 (14.7)	14 (34.1)	
2	0 (0)	2 (4.9)	
Location			0.220
Anterior	24 (22.0)	13 (31.7)	
Posterior	85 (78.0)	28 (68.3)	
D&E	25 (22.9)	12 (29.3)	0.556
Resectoscope	9 (8.3)	1 (2.4)	0.365
IVF	36 (33.0)	12 (29.3)	0.808
Myomectomy	5 (4.6)	4 (9.8)	0.422
**Maternal outcomes**			
Bleeding during pregnancy	35 (32.1)	16 (39.0)	0.546
EBL (mL)	958.3 ± 528.9	1627.8 ± 962.8	< 0.001
Pre-post Hb difference (g/dL)	2.4 ± 1.2	3.0 ± 1.4	0.009
Transfusion requirement	41 (37.6)	23 (56.0)	0.041
Transfusion (packs)^*^	0.9 ± 1.6	2.5 ± 3.5	0.008
Intrauterine balloon tamponade	61 (56.0)	21 (51.2)	0.737
Uterine artery embolization	7 (6.4)	6 (14.6)	0.205
Hysterectomy	1 (0.9)	13 (31.7)	< 0.001
ICU admission	1 (0.9)	5 (12.2)	0.007
**Neonatal outcomes**			
Preterm birth			
GA < 37 weeks	30 (27.5)	21 (51.2)	0.006
GA < 34 weeks	3 (2.8)	4 (9.8)	0.089
Birth weight (g)	2911.3 ± 354.9	2787.1 ± 551.7	0.186
Birthweight < 2,500 g	15 (13.8)	12 (29.3)	0.028
NICU admission	24 (22.0)	16 (39.0)	0.036
Ventilatory support (intubation)	13 (11.9)	10 (24.4)	0.059
SGA	9 (8.3)	7 (17.1)	0.141
1-minute AS < 7	60 (55.0)	31 (75.6)	0.022
5-minute AS < 7	4 (3.7)	9 (22.0)	0.001

Data are presented as mean ± standard deviation or number (percentage).

* Number of packed red blood cell (RBC) units transfused.

Hb, hemoglobin; CS, caesarean section; AS, Apgar score; GA, gestational age; SGA, small for gestational age; PAS, placenta accreta spectrum; SD, standard deviation; IVF, *in vitro* fertilization; NICU, Neonatal intensive care unit; EBL, Estimated blood loss; D&E, Dilation and evacuation.

**Fig 1:**
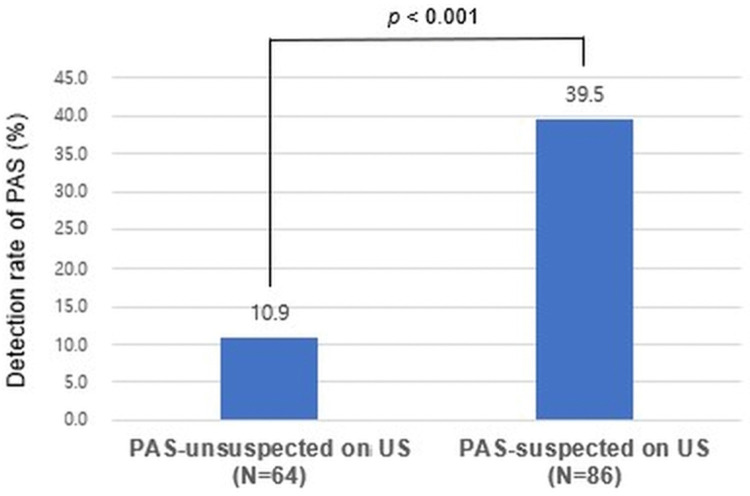
Detection rate of placenta accreta spectrum (PAS) on prenatal ultrasonography (US). The detection rate of confirmed PAS was significantly higher in PAS-suspected on US group than‌‌ PAS-unsuspected on US group (39.5% vs 10.9%, *p* < 0.001). PAS, placenta accreta spectrum‌‌; US, ultrasonography.

US demonstrated significant diagnostic value in detecting PAS and in predicting maternal and neonatal outcomes. Prenatal suspicion of PAS (hereafter referred to as the PAS-suspected on US group) were significantly associated with a confirmed diagnosis of PAS (39.5% vs 10.9%, *p* < 0.001) (Fig 1). Regarding clinical characteristics, previa type, history of previous caesarean section (29.1% vs 10.9%, *p* = 0.003) and history of myomectomy (10.5% vs 0.0%, *p* = 0.010) showed significant differences between PAS-suspected on US group and PAS-unsuspected on US group. Additionally, regarding maternal and neonatal outcomes, PAS-suspected on US group also exhibited significantly higher EBL (1323.1 ± 844.0 mL vs 896.9 ± 461.6 mL, *p* < 0.001), transfusion requirement (55.8% vs 25.0%, *p* < 0.001), number of transfusion units administered (1.8 ± 2.7 vs 0.7 ± 1.7, *p* = 0.003), use of intrauterine balloon tamponade (65.1% vs 40.6%, *p* = 0.003), incidence of hysterectomy (15.1% vs 1.6%, *p* = 0.005) and preterm birth before 37 weeks of gestation (47.7% vs 15.6%, *p* < 0.001), neonatal ventilatory support (20.9% vs 7.8%, *p* = 0.027), 1-minute AS < 7 (69.8% vs 48.4%, *p* = 0.008) than the group without prenatal suspicion of PAS on US (hereafter referred to as the PAS-unsuspected on US group) (Table 2).

**Table 2:** Prediction of maternal and neonatal outcomes by US.

	PAS-unsuspected on US (*n* = 64)	PAS-suspected on US (*n* = 86)	*p*-value
**Clinical characteristics**			
Age (years)	36.0 ± 3.5	36.1 ± 4.1	0.914
GA at delivery (weeks)	36.9 ± 1.1	36.4 ± 1.4	0.008
Previa (type)			0.045
Complete	31 (48.4)	61 (70.9)	
Partial	9 (14.1)	7 (8.1)	
Marginal	13 (20.3)	11 (12.8)	
Low-lying	11 (17.2)	7 (8.1)	
Previous CS	7 (10.9)	25 (29.1)	0.007
Location			0.494
Anterior	14 (21.9)	23 (26.7)	
Posterior	50 (78.1)	63 (73.3)	
D&E	16 (25.0)	21 (24.4)	0.935
Resectoscope	5 (7.8)	5 (5.8)	0.745
IVF	24 (37.5)	24 (27.9)	0.213
Myomectomy	0 (0.0)	9 (10.5)	0.010
**Maternal outcomes**			
EBL (mL)	896.9 ± 461.6	1323.1 ± 844.0	<0.001
Pre-post Hb difference (g/dL)	2.5 ± 1.2	2.7 ± 1.3	0.404
Transfusion requirement	16 (25.0)	48 (55.8)	<0.001
Transfusion (packs)^*^	0.7 ± 1.7	1.8 ± 2.7	0.003
Intrauterine balloon tamponade	26 (40.6)	56 (65.1)	0.003
Uterine artery embolization	3 (4.7)	10 (11.6)	0.135
Hysterectomy	1 (1.6)	13 (15.1)	0.005
ICU admission	1 (1.6)	5 (5.8)	0.240
**Neonatal outcomes**			
Preterm birth			
GA < 37 weeks	10 (15.6)	41 (47.7)	<0.001
GA < 34 weeks	1 (1.5)	6 (7.0)	0.239
Birth weight (g)	2938.3 ± 364.2	2832.0 ± 453.4	0.125
Birthweight < 2,500 g	9 (14.1)	18 (20.9)	0.279
SGA	7 (10.9)	9 (10.5)	0.926
NICU admission	14 (21.9)	26 (30.2)	0.252
Ventilatory support (intubation)	5 (7.8)	18 (20.9)	0.027
1-minute AS < 7	31 (48.4)	60 (69.8)	0.008
5-minute AS < 7	4 (6.3)	9 (10.5)	0.364

Data are presented as mean ± standard deviation or number (percentage).

* Number of packed red blood cell units transfused.

Hb, hemoglobin; AS, Apgar score; GA, gestational age; SGA, small for gestational age; ICU, intensive care unit; EBL, estimated blood loss; NICU, neonatal intensive care unit; PAS, placenta accreta spectrum; SD, standard deviation; US, ultrasonography.

**Fig 2:**
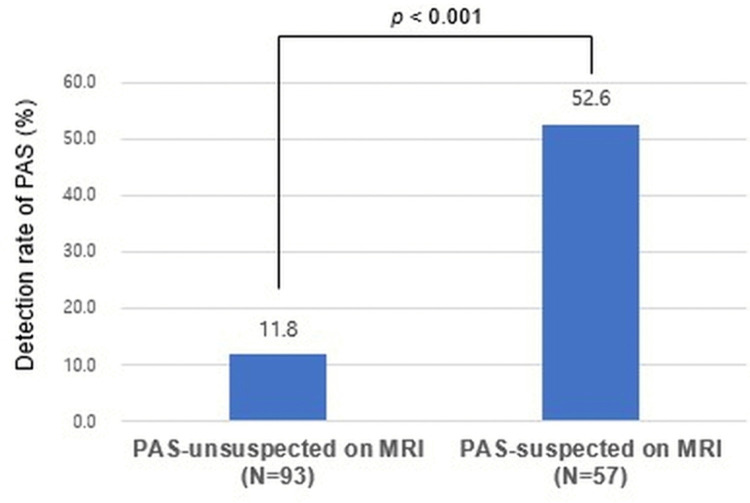
Detection rate of placenta accreta spectrum (PAS) on prenatal magnetic resonance imaging (MRI). The rate of confirmed PAS was markedly higher in PAS-suspected on MRI group than in PAS-unsuspected on MRI group (52.6% vs 11.8%, *p* < 0.001), indicating a significant diagnostic association. PAS, placenta accreta spectrum; MRI, magnetic resonance imaging.

Fig 2 illustrates that prenatal suspicion of PAS on MRI (hereafter referred to as the PAS-suspected on MRI group) strongly correlated with the confirmed diagnosis of PAS (52.6% vs 11.8%, *p* < 0.001). The number of transfusion units administered (2.2 ± 3.3 vs 0.8 ± 1.25, *p* = 0.005), incidence of hysterectomy (22.8% vs 1.1%, *p* < 0.001), maternal ICU admission (10.5% vs 0%, *p* = 0.003), preterm birth before 37 weeks of gestation (49.1% vs 23.9%, *p* = 0.002) and before 34 weeks of gestation (10.5% vs 1.1%, *p* = 0.012), neonatal ICU (NICU) admission (36.8% vs 20.4%, *p* = 0.027), neonatal ventilatory support (26.3% vs 8.6%, *p* = 0.003), 1-minute AS < 7 (73.7% vs 52.7%, *p* = 0.011), and 5-minute AS < 7 (15.8% vs 4.3%, *p* = 0.032) occurred more frequently in the PAS-suspected on MRI group than in the group without prenatal suspicion of PAS on MRI (hereafter referred to as the PAS-unsuspected on MRI group) (Table 3).

**Table 3:** Prediction of maternal and neonatal outcomes by MRI.

	PAS-unsuspected on MRI (*n* = 93)	PAS-suspected on MRI (*n* = 57)	*p*-value
**Clinical characteristics**			
Age (years)	36.4 ± 4.0	35.6 ± 3.6	0.208
GA at delivery (weeks)	36.9 ± 1.1	36.2 ± 1.5	0.003
Previa (type)			0.211
Complete	51 (54.8)	41 (71.9)	
Partial	11 (11.8)	5 (8.8)	
Marginal	18 (19.4)	6 (10.5)	
Low-lying	13 (14.0)	5 (8.8)	
Previous CS	16 (17.2)	16 (28.1)	0.115
Location			0.449
Anterior	21 (22.6)	16 (28.1)	
Posterior	72 (77.4)	41 (71.9)	
D&E	21 (22.6)	16 (28.1)	0.449
Resectoscope	8 (8.6)	2 (3.5)	0.319
IVF	33 (35.5)	15 (26.3)	0.243
Myomectomy	4 (4.3)	5 (8.8)	0.301
**Maternal outcomes**			
EBL (mL)	1042.9 ± 557.4	1301.8 ± 942.0	0.063
Pre-post Hb difference (g/dL)	2.6 ± 1.2	2.6 ± 1.3	0.841
Transfusion requirement	36 (38.7)	28 (49.1)	0.211
Transfusion (packs)*	0.8 ± 1.2	2.2 ± 3.3	0.005
Intrauterine balloon tamponade	49 (52.7)	33 (57.9)	0.534
Uterine artery embolization	5 (5.4)	8 (14.0)	0.079
Hysterectomy	1 (1.1)	13 (22.8)	< 0.001
ICU admission	0 (0)	6 (10.5)	0.003
**Neonatal outcomes**			
Preterm birth			
GA< 37 weeks	22 (23.9)	28 (49.1)	0.002
GA< 34 weeks	1 (1.1)	6 (10.5)	0.012
Birth weight (g)	2929.5 ± 367.1	2792.3 ± 485.1	0.069
Birthweight < 2,500 g	13 (14.0)	14 (24.6)	0.102
SGA	8 (8.6)	8 (14.0)	0.295
NICU admission	19 (20.4)	21 (36.8)	0.027
Ventilatory support (intubation)	8 (8.6)	15 (26.3)	0.003
1-minute AS < 7	49 (52.7)	42 (73.7)	0.011
5-minute AS < 7	4 (4.3)	9 (15.8)	0.032

Data are presented as mean ± standard deviation or number (percentage).

* Number of packed red blood cell units transfused.

Hb, hemoglobin; AS, Apgar score; GA, gestational age; SGA, small for gestational age; ICU, intensive care unit; EBL, estimated blood loss; NICU, neonatal intensive care unit; PAS, placenta accreta spectrum; SD, standard deviation; MRI, magnetic resonance imaging.

The diagnostic performances of US and MRI for detecting PAS are presented in Table 4. US demonstrated a sensitivity of 82.9%, specificity of 52.3%, PPV of 39.5%, NPV of 89.1%, and overall diagnostic accuracy of 60.7%. In comparison, MRI exhibited a sensitivity of 73.2%, specificity of 75.2%, PPV of 52.6%, NPV of 88.2%, and an accuracy of 74.7%. Although a significant overall discrepancy was observed in the PAS-suspected on between US and MRI (*n* = 150, *p* = 0.013 by McNemar’s test), subgroup analyses revealed no significant differences in sensitivity among patients diagnosed with PAS cases (*n* = 41, *p* = 0.270 by McNemar’s test) and specificity among cases diagnosed without PAS (*n* = 109, *p* = 0.619 by McNemar’s test). This suggests that the overall difference was driven by the distribution of discordant classifications rather than a consistent superiority of one modality over the other in either sensitivity or specificity. Based on these findings, the diagnostic performance of combined US and MRI for the detection of PAS was further evaluated.

**Table 4:** Sensitivity, specificity, PPV, NPV, and accuracy of US and MRI in detecting PAS.

	Sensitivity (%)	Specificity (%)	PPV (%)	NPV (%)	Accuracy (%)
**US**	82.9	52.3	39.5	89.1	60.7
**MRI**	73.2	75.2	52.6	88.2	74.7
**US + MRI**	63.4	87.2	65.0	86.4	80.7

PPV, positive predictive value; NPV, negative predictive value; US, ultrasonography; MRI, magnetic resonance imaging; PAS, placenta accreta spectrum.

**Fig 3:**
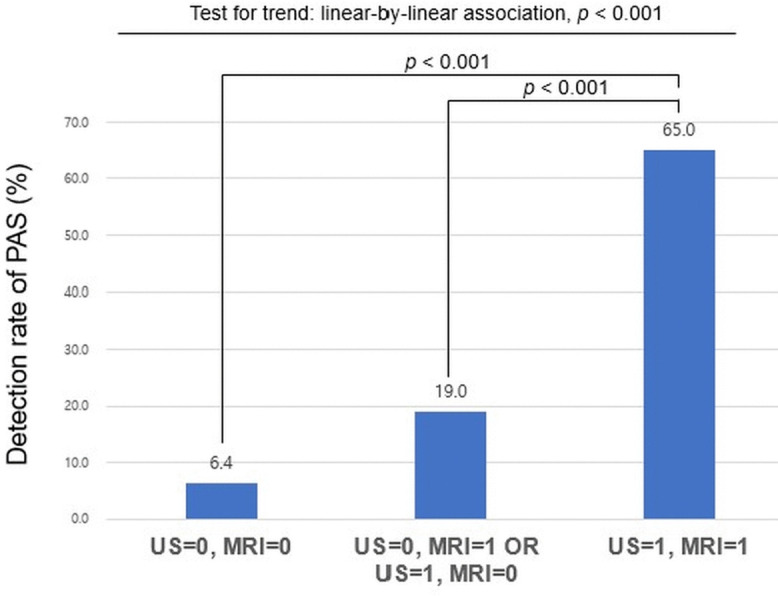
Final diagnosis of placenta accreta spectrum (PAS). The final diagnosis was made with a combination of US and MRI. Patients were categorized into three groups based on prenatal imaging results: 1) both US and MRI findings were negative (US = 0, MRI = 0); 2) either US or MRI was positive, but not both (US = 0, MRI = 1 or US = 1, MRI = 0); and 3) both US and MRI were positive (US = 1, MRI = 1). A significant difference was observed between the group with US = 0 and MRI = 0 and the group with US = 1 and MRI = 1 (*p* < 0.001), as well as between the single-positive group (US = 0 and MRI = 1 or US = 1 and MRI = 0) and the group with US = 1 and MRI = 1 (*p* < 0.001). A significant linear trend was noted in PAS occurrence across the three diagnostic combinations (*p* < 0.001), with the highest rate observed in cases positive on both US and MRI. PAS, placenta accreta spectrum; US, ultrasonography; MRI, magnetic resonance imaging.

After categorizing patients into three groups according to combined radiological findings of US and MRI, the following observations were made: when US and MRI findings were negative (US = 0, MRI = 0), PAS was confirmed in only 3 out of 47 cases (6.4%); in cases in which either US or MRI was positive, but not both, 12 out of 63 cases (19.0%) were diagnosed with PAS; when US and MRI were positive (US = 1, MRI = 1), PAS was confirmed in 26 out of 40 cases (65.0%). A statistically significant difference was observed among the groups (*p <* 0.001). In addition, a statistically significant linear trend was identified among these three groups and PAS diagnosis (*p* < 0.001 by the analysis of linear-by-linear association) (Fig 3). The predictive performance of US + MRI to detect PAS, when dichotomized using Youden’s index, is presented in Table 4.

**Fig 4:**
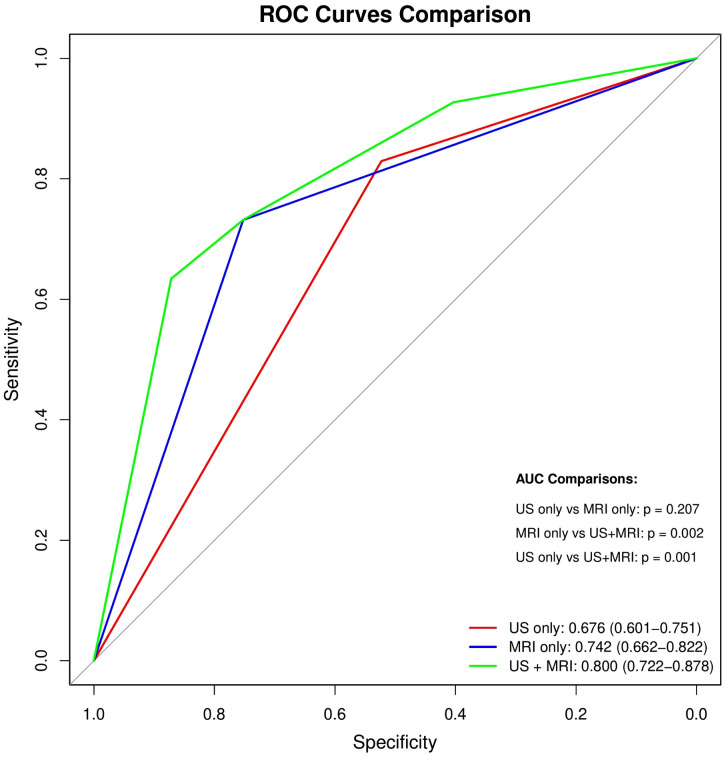
Receiver operating characteristic (ROC) curve comparison for placenta accreta spectrum (PAS) diagnosis. Patients were diagnosed using US-only, MRI-only, and US + MRI models. The ROC curves demonstrate the diagnostic performance of US alone (red), MRI alone (blue), and a combination of US and MRI (green) in detecting PAS. The combined model showed the highest overall diagnostic accuracy, with a greater AUC than each modality alone. Pairwise comparisons demonstrated statistically significant differences between US-only and MRI-only (*p* = 0.207), MRI-only and US + MRI (*p* = 0.002), and US-only and US + MRI (*p* = 0.001) cases. AUC, area under the curve; MRI, magnetic resonance imaging; PAS, placenta accreta spectrum; ROC, receiver operating characteristic; US, ultrasonography.

A ROC curve analysis was subsequently performed to compare the diagnostic performance of US alone, MRI alone, and US + MRI for the detection of PAS. As illustrated in Fig 4, the AUC was highest when US and MRI were used in combination, indicating superior overall diagnostic accuracy. DeLong’s tests for differences in AUC revealed statistically significant differences between MRI-only and US + MRI (*p* = 0.002) and between US-only and US + MRI (*p* = 0.001) cases. The combined US + MRI model outperformed both US and MRI alone, suggesting that integration of both modalities may enhance diagnostic performance in the detection of PAS.

The predictive performance of the combined US and MRI findings for maternal and neonatal outcomes is presented in Table 5. Maternal morbidity demonstrated a linear trend of increase across the four groups stratified by US and MRI findings, with the highest morbidity observed when both modalities indicated suspicion of PAS. EBL (*p* = 0.006), incidence of transfusion requirement (*p* = 0.001), and number of transfusion units administered (*p* < 0.001), use of intrauterine ballon tamponade (*p* = 0.020), uterine artery embolization (*p* = 0.033), hysterectomy (*p* < 0.001), and maternal ICU admission (*p* = 0.004) showed a significant increasing trend across the three groups. Neonatal outcomes also deteriorated in association with combined US and MRI findings. Preterm birth before 37 weeks of gestation (*p* < 0.001), NICU admission (*p* = 0.032), neonatal ventilatory support (*p* = 0.001), and 1-minute AS < 7 (*p* < 0.001), and 5-minute AS < 7 (*p* = 0.033) had significant linear increases towards the US = 1/MRI = 1 group.

**Table 5:** Prediction of maternal and neonatal outcomes based on US and MRI combination.

	US = 0, MRI = 0 (*n* = 47)	US = 1, MRI = 0 or US = 0, MRI = 1 (*n* = 63)	US = 1, MRI = 1 (*n* = 40)	*p*-value
**Maternal outcomes**				
EBL (mL)	866.0 ± 428.6	1148.2 ± 604.7	1437.5 ± 1042.9	0.006^†^
Pre-post Hb difference (g/dL)	2.6 ± 1.2	2.6 ± 1.4	2.8 ± 1.4	0.780^†^
Transfusion requirement	10 (21.3)	32 (50.8)	22 (55.0)	0.001^‡^
Transfusion (packs)^*^	0.6 ± 1.2	1.6 ± 2.1	3.3 ± 4.1	<0.001^†^
Intrauterine balloon tamponade	16 (34.0)	43 (68.3)	23 (57.5)	0.020^‡^
Uterine artery embolization	1 (2.1)	6 (9.5)	6 (15.0)	0.033^‡^
Hysterectomy	0 (0.0)	2 (3.2)	12 (30.0)	<0.001^‡^
ICU admission	0 (0.0)	1 (1.6)	5 (12.5)	0.004^‡^
**Neonatal outcomes**				
Preterm birth				
GA < 37 weeks	6 (12.8)	21 (33.3)	24 (60.0)	<0.001^‡^
Birth weight (g)	2948.7 ± 382.6	2928.4 ± 354.9	2728.3 ± 525.4	0.116^†^
Birthweight < 2,500 g	7 (14.9)	8 (12.7)	12 (30.0)	0.081^‡^
SGA	4 (8.5)	7 (11.1)	5 (12.5)	0.545^‡^
NICU admission	11 (23.4)	11 (17.5)	18 (45.0)	0.032^‡^
Ventilatory support (intubation)	3 (6.4)	7 (11.1)	13 (32.5)	0.001^‡^
1-minute AS < 7	22 (46.8)	36 (57.1)	33 (82.5)	<0.001^‡^
5-minute AS < 7	3 (6.4)	2 (3.2)	8 (20.0)	0.033^‡^

Data are presented as mean ± standard deviation or number (percentage).

* Number of packed red blood cell units transfused.

† Jonckheere–Terpstra test (for continuous variables); ‡ Linear-by-linear association (for categorical variables).

Hb, hemoglobin; AS, Apgar score; GA, gestational age; EBL, estimated blood loss; SGA, small for gestational age; ICU, intensive care unit; NICU, neonatal intensive care unit; PAS, placenta accreta spectrum; SD, standard deviation.

To show the usefulness of US and MRI findings in prediction of perinatal outcomes, subgroup analysis was conducted among patients without diagnosed PAS and those with diagnosed. Among patients without diagnosed PAS, PAS-suspected on US group demonstrated significantly higher incidence of transfusion requirement (50.0% vs. 26.3%, *p* = 0.011), use of intrauterine balloon tamponade (75.0% vs. 38.6%, *p* < 0.001), and preterm birth before 37 weeks of gestation (42.3% vs. 14.0%, *p* < 0.001) than PAS-unsuspected on US group (S1 Table). EBL and incidence of transfusion requirement showed significantly higher in PAS-suspected on US group among patients with diagnosed PAS (S2 Table). Among women without diagnosed PAS, PAS-suspected on MRI group did not show statistically significant associations with most maternal outcomes. Although hysterectomy and ICU admission occurred only in PAS-suspected on MRI group, the differences were not statistically significant (*p* = 0.080 for both) (S3 Table). Neonatal outcomes were likewise similar between the two groups, but NICU admission and ventilatory support tended to be more frequent in PAS-suspected on MRI group (33.3% vs. 18.3% and 22.2% vs. 8.5%, respectively), but these differences did not reach statistical significance (S3 Table).

Among patients with diagnosed PAS, PAS-suspected on MRI group was associated with more severe maternal morbidity. Although EBL was higher in PAS-suspected on MRI group (1736.7 ± 1046.4 mL vs. 1330.9 ± 631.0 mL), the difference did not reach statistical significance. However, the number of transfusion units administered and preterm birth before 37 weeks of gestation was significantly greater in PAS-suspected on MRI group (3.2 ± 3.8 vs. 0.6 ± 0.8, *p* = 0.002). Incidence of hysterectomy (40.0% vs. 9.1%) and maternal ICU admission (16.7% vs. 0%) were numerically higher among PAS-suspected on MRI group, although statistical significance was not achieved (S4 Table).

## Discussion

This study demonstrates that both US and MRI are useful to predict adverse maternal and neonatal outcomes as well as to detect PAS. In detecting PAS, US and MRI demonstrated significant diagnostic value (Figs 1 and 2). In predicting perinatal outcomes, US, but not MRI, is informative for predicting higher EBL, transfusion requirement, and use of intrauterine balloon tamponade, whereas MRI, but not US, predicts maternal ICU admission, preterm birth before 34 weeks of gestation, NICU admission, and 5-minute AS <7. The combined use of US and MRI findings demonstrated a progressively higher detection rate of PAS (Fig 3) and prediction rate of adverse perinatal outcomes across stratified groups (Table 5), underscoring the complementary strengths of these imaging modalities in clinical decision-making. Furthermore, even in the absence of PAS, PAS-suspected imaging findings on US or MRI were helpful in predicting adverse perinatal outcomes.

We adopted a diagnostic threshold (at least ≥ 2 US indicators). In earlier studies published more than a decade ago, a threshold of ≥ 1 US finding was often used for analysis [18,35]. However, recent studies and professional societies have advocated considering multiple imaging features, typically ≥ 2 findings, to improve the prenatal prediction of placenta accreta spectrum (PAS) [29,36].

**Fig 5:**
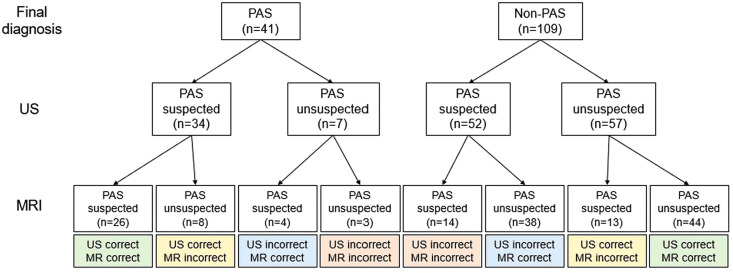
Flowchart comparing the diagnostic concordance between US and MRI. Patients were classified by prenatal US suspicion and then by MRI suspicion, with each terminal box indicating whether US or MRI were correct relative to the final diagnosis confirmed postnatally. PAS, placenta accreta spectrum; US, ultrasonography; MRI, magnetic resonance imaging.

Our data support the combined use of US and MRI, particularly in patients with ambiguous or borderline imaging findings. Fig 5 illustrates the variable diagnostic concordance between the two modalities. Cases in which both US and MRI correctly identified either the presence or absence of PAS (*n* = 70) exhibited optimal diagnostic agreement. However, a considerable proportion of cases demonstrated discordant findings, in which only one modality provided the correct diagnosis (e.g., US correct/MRI incorrect in *n* = 21, and MRI correct/US incorrect in *n* = 42). These findings underscore the inherent limitations of relying on a single imaging modality, particularly in diagnostically challenging cases. US and MRI contribute different strengths to the evaluation of PAS. Understanding the complementary roles of US and MRI helps to explain this difference in diagnostic performance between US and MRI. US allows rapid bedside assessment of key diagnostic markers and is particularly useful for visualizing vascular patterns within the affected region, a feature that MRI does not capture as effectively. In contrast, MRI provides superior definition of soft tissues, facilitating a clearer evaluation of adhesions and the depth or extent of myometrial involvement. When used together, the two techniques function in a complementary manner: US enhances accessibility and vascular assessment, whereas MRI offers detailed anatomical characterization that can better inform surgical planning. Therefore, final clinical decision-making for suspected PAS should be based on an integrated assessment incorporating US findings, MRI findings, clinical risk factors, and multidisciplinary evaluation, rather than reliance on a single imaging modality. Representative cases based imaging findings according to concordant and discordant diagnostic groups, US-positive/MRI-positive (US = 1, MRI = 1), US-positive/MRI-negative (US = 1, MRI = 0), and US-negative/MRI-positive (US = 0, MRI = 1), are provided in S1–S3 Figs.

Regarding perinatal outcomes, the predictive value of US and MRI has not yet been firmly established, and studies evaluating their utility for neonatal outcomes in particularly remain limited. A notable strength of our research is that we assessed maternal and neonatal outcomes for both US and MRI findings. According to Del Negro, et al. more pronounced US features suggestive of PAS were associated with earlier gestational age at delivery and lower AS [37]. This finding implies a correlation between severe US features and adverse neonatal outcomes at birth. However, in our study, although earlier delivery NICU admission rates and 5-minute AS <7 did not differ significantly in PAS-suspected on US group. With respect to MRI, Delli Pizzi A, et al. [26] and Charis Bourgioti, et al. [33] reported that MRI findings were significantly associated with adverse maternal outcomes; however, they showed no meaningful differences in neonatal outcomes. In contrast, our study identified significant differences not only in maternal complications, such as hysterectomy and maternal ICU admission, but also in several neonatal events, including preterm birth, NICU admission, neonatal ventilatory support, and low AS in PAS-suspected on MRI group. Our findings also demonstrate that the combined use of US and MRI improves risk stratification for PAS, correlating with increased maternal and neonatal morbidities. These results suggest that a combination of US and MRI findings may serve as a predictor of adverse perinatal outcomes, highlighting the value of integrating US and MRI findings in prenatal assessments.

Beyond diagnostic performance, our findings highlight that imaging characteristics themselves carry important implications for perinatal outcomes. Among patients who were not diagnosed with PAS, prenatal imaging on US revealed that those who were PAS-suspected on US group showed significantly higher incidence of transfusion requirement, use of intrauterine balloon tamponade, and preterm birth before 37 weeks of gestation than those who were PAS-unsuspected on US group (S1 Table). In contrast, MRI-based categorization did not demonstrate significant differences in perinatal outcomes within this same group, not diagnosed with PAS (S3 Table).

Interestingly, the pattern differed among patients in the diagnosed PAS group; individuals who were PAS-suspected on US group showed significantly higher EBL and incidence of transfusion requirement (S2 Table); individuals who were PAS-suspected on MRI group significantly higher number of transfusion units administered and incidence of preterm birth before 37 weeks of gestation than those who were PAS-unsuspected on MRI group (S4 Table).

In current study, we included only women with placenta previa, as this condition represents the most clinically relevant risk factor for PAS and is associated with higher maternal and neonatal morbidity. Although PAS can occur in the absence of placenta previa, these cases are generally associated with lower clinical severity, a reduced need for surgical intervention, and decreased maternal blood loss compared to PAS with placenta previa [38]. Risk factor also differ between PAS with placenta previa and PAS without placenta previa, the latter is more commonly associated with dilation and curettage, operative hysteroscopic procedures, adenomyosis, and assisted reproductive technologies [39,40]. A possible explanation for the differing risk factors may lie in the anatomical location of endometrial injury. Scarring from caesarean sections typically involves the lower uterine segment, which overlaps with the primary implantation site in placenta previa, whereas intrauterine procedures are more likely to disrupt the upper uterine segment, thereby increasing the likelihood of abnormal placental adherence in the absence of placenta previa [41]. Although, PAS without placenta previa was not included in the present study, pregnant women with associated risk factors still necessitate heightened clinical vigilance and comprehensive prenatal assessment to mitigate the risk of serious maternal outcomes.

This study has several strengths. First, it provides a comprehensive evaluation of US and MRI findings in the same patient cohort, covering the entire spectrum of placenta previa, from low-lying to complete cases. Unlike previous studies that focused on patients with a high clinical suspicion of PAS, this study benefited from a broader inclusion of cases, allowing for a more balanced and objective assessment of the diagnostic performance of both modalities. All patients diagnosed with placenta previa underwent MRI, facilitating a systematic comparison of US and MRI findings across a wide range of clinical presentations. Second, this study was conducted at a high-volume single tertiary care center with substantial experience in managing PAS. The center performs more than 3,000 deliveries for high-risk pregnancies annually, ensuring a robust clinical setting for evaluating complex obstetric conditions. Third, to minimize diagnostic bias, all imaging data were meticulously reviewed by a multidisciplinary team comprising obstetric specialists and radiologists with expertise in PAS diagnosis, independent of clinical information. This high-level expert review ensures greater accuracy and reliability in image interpretation, reduces inter-observer variability, and reinforces the validity of the study’s findings. Furthermore, in comparison with another study that included only 42 pregnant women who underwent both US and MRI [18], our study examined a substantially larger cohort of 150 patients. Lastly, we evaluated not only the detection of PAS but also the prediction of perinatal outcomes. Notably, even in cases suspected of PAS on US or MRI but ultimately not diagnosed PAS, perinatal outcomes remained poor.

Our study also has some limitations. First, among histopathologically confirmed PAS cases, most patients were diagnosed with placenta accreta, whereas only two patients were diagnosed with placenta increta, accounting for 15.4% (2/13) of cases; no cases of placenta percreta were observed. As a result, US signs associated with increta and percreta, such as abnormal uterine contours, exophytic masses, and bridging vessels [28], were absent, which may have contributed to the lower diagnostic performance of US. Second, only two patients with more than two prior caesarean sections, which is one of the key risk factors for PAS [4,42,43], were included. This may have reduced the statistical power of the analysis. Third, as this study had a retrospective design, it relied on existing medical records and imaging data, including ultrasound images with inherent limitations, which may be incomplete or subject to inaccuracies. However, the current study was conducted at a high-volume, single tertiary care center, where all clinicians are experienced specialists routinely managing high-risk pregnancies. As a result, clinical practice is highly standardized, with minimal interobserver variability in the diagnosis of PAS. Lastly, formal inter-reader agreement could not be assessed because independent preliminary interpretations were not systematically recorded; instead, all imaging interpretations were finalized by consensus.

Further research is needed to optimize the diagnostic accuracy of PAS through the integrated analysis of detailed US and MRI features, to develop a robust predictive model, given their critical implication for perinatal risk assessment and surgical planning. Multicenter, large-scale, prospective studies are warranted to validate these findings and to generalize the applicability of our results.

## Conclusions

US and MRI significantly enhanced the detection of PAS and its associated adverse perinatal outcomes in women with placenta previa. Importantly, the substantial proportion of discordant cases between the two modalities underscores the limitations of relying on a single imaging technique. US, but not MRI, is informative for predicting higher EBL, transfusion requirement, and use of intrauterine balloon tamponade, whereas MRI, but not US, predicts maternal ICU admission, preterm birth before 34 weeks of gestation, NICU admission, and 5-minute AS <7. Our findings suggest that the combined use of US and MRI improves diagnostic reliability, particularly in equivocal cases. In addition, combining both imaging modalities may contribute to the prediction of adverse perinatal outcomes, including maternal hemorrhage and neonatal complications. Further investigation should focus on integrating detailed US and MRI features to enhance the detection of PAS and adverse perinatal outcomes.

## Supporting information

S1 Table Perinatal outcomes based on US findings in patients without diagnosed PAS.
(DOCX)

S1 Fig Concordant imaging findings (US-positive/MRI-positive) in Placenta accreta case 1.
(A) Transabdominal gray-scale US shows multiple lacunae in placenta (arrow). (B) Transvaginal color Doppler US image shows uterine serosa-bladder interface hypervascularity. (C) Sagittal T2-weighted MRI reveal multiple T2-dark bands (arrow) and marked intraplacental vascularity in the lower uterine segment (arrowhead). The patient was clinically diagnosed with PAS. US, ultrasonography; MRI, magnetic resonance imaging; PAS, placenta accreta spectrum.
(TIF)

S2 Table Perinatal outcomes based on US findings in patients with diagnosed PAS.
(DOCX)

S2 Fig Discordant imaging findings (US-positive/MRI-negative) case in a normal placenta.
A 29-year-old woman presented with three US findings highly suggestive of PAS; however, were not corroborated by MRI, and the final diagnosis confirmed a normal placenta (A) Transvaginal color Doppler US showing subplacental hypervascularity. (B) Transabdominal gray-scale US shows multiple lacunae (arrowhead). (C) Transvaginal gray-scale US shows loss of clear zone (arrow). (D) Sagittal T2-weighted MRI shows no imaging features suggestive of placenta accreta. US, ultrasonography; MRI, magnetic resonance imaging.
(TIF)

S3 Table Perinatal outcomes based on MRI findings in patients without diagnosed PAS.
(DOCX)

S3 Fig Discordant imaging findings (US-negative/MRI-positive) case in a normal placenta.
(A) Transabdominal gray-scale US showing no placental lacunae. (B) Transvaginal color Doppler US shows no signs of subplacental hypervascularity. (C) Sagittal T2-weighted MRI shows multiple T2-dark bands (arrow) suggestive of placenta accreta. US, ultrasonography; MRI, magnetic resonance imaging.
(TIF)

S4 Table Perinatal outcomes based on MRI findings in patients with diagnosed PAS.
(DOCX)
