# Supplementary material for: Detection of placenta accreta spectrum and prediction of adverse perinatal outcomes in pregnant women with placenta previa using ultrasonography and magnetic resonance imaging: A retrospective cohort study
Source: PLoS One. 2026 May 29;21(5):e0349503. doi: 10.1371/journal.pone.0349503 (PMC13221029; doi:10.1371/journal.pone.0349503)
Supplement: S1 Table — Perinatal outcomes based on US findings in patients without diagnosed PAS. (DOCX) [file pone.0349503.s001.docx]

**S1 Table.** Perinatal outcomes based on US findings in patients without diagnosed PAS

|  | **PAS-unsuspected on US (*n* = 57)** | **PAS-suspected**  **on US (*n* = 52)** | ***p*-value** |
| --- | --- | --- | --- |
| **Maternal outcomes** |  |  |  |
| EBL (mL) | 870.2 ± 463.0 | 1054.8 ± 582.0 | 0.072 |
| Pre-post Hb difference | 2.4 ± 1.1 | 2.5 ± 1.2 | 0.604 |
| Transfusion requirement | 15 (26.3) | 26 (50.0) | 0.011 |
| Transfusion (packs)^*^ | 0.7 ± 1.7 | 1.1 ± 1.4 | 0.209 |
| Intrauterine balloon tamponade | 22 (38.6) | 39 (75.0) | <0.001 |
| Uterine artery embolization | 2 (3.5) | 5 (9.6) | 0.255 |
| Hysterectomy | 1 (1.8) | 0 (0.0) | 1.000 |
| ICU admission | 1 (1.8) | 0 (0.0) | 1.000 |
| **Neonatal outcomes** |  |  |  |
| Preterm birth |  |  |  |
| GA < 37 weeks | 8 (14.0) | 22 (42.3) | <0.001 |
| Birth weight (g) | 2921.8 ± 354.8 | 2899.8 ± 358.2 | 0.749 |
| Birthweight < 2,500 g | 8 (14.0) | 7 (13.5) | 0.931 |
| SGA | 6 (10.5) | 3 (5.8) | 0.493 |
| NICU admission | 12 (21.1) | 12 (23.1) | 0.799 |
| Ventilatory support (intubation) | 5 (8.8) | 8 (15.4) | 0.287 |
| 1-minute AS < 7 | 27 (47.4) | 33 (63.5) | 0.092 |
| 5-minute AS < 7 | 3 (5.3) | 1 (1.9) | 0.620 |

Data are presented as mean ± standard deviation or number (percentage).

^*^Number of packed red blood cell units transfused.

Hb, hemoglobin; AS, Apgar score; GA, gestational age; SGA, small for gestational age; ICU, intensive care unit; EBL, estimated blood loss; NICU, neonatal intensive care unit; PAS, placenta accreta spectrum; SD, standard deviation; US, ultrasonography.
