# Supplementary figures and images for: Detection of placenta accreta spectrum and prediction of adverse perinatal outcomes in pregnant women with placenta previa using ultrasonography and magnetic resonance imaging: A retrospective cohort study
Source: PLoS One. 2026 May 29;21(5):e0349503. doi: 10.1371/journal.pone.0349503 (PMC13221029; doi:10.1371/journal.pone.0349503)

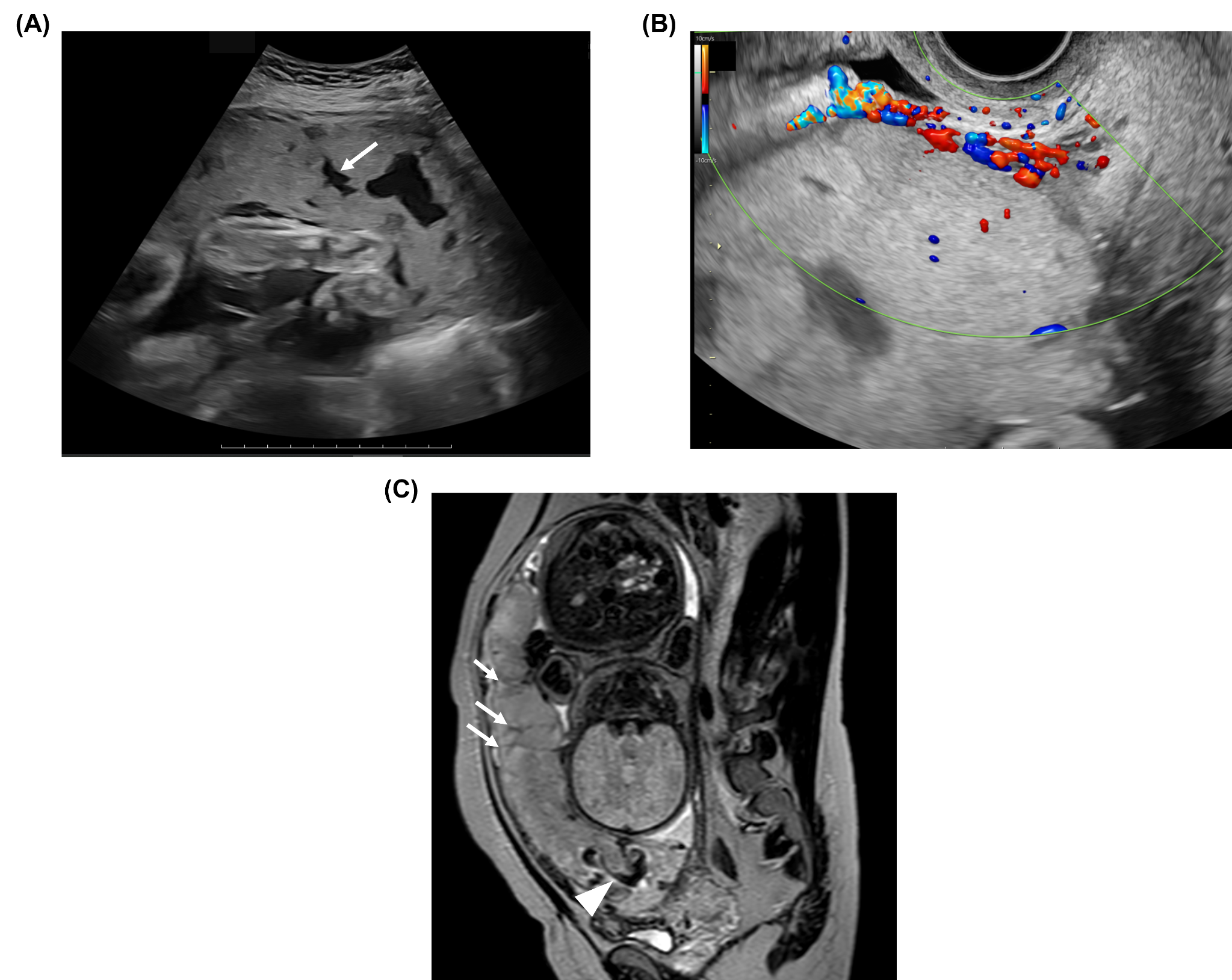

Supplement: S1 Fig — Concordant imaging findings (US-positive/MRI-positive) in Placenta accreta case 1. (A) Transabdominal gray-scale US shows multiple lacunae in placenta (arrow). (B) Transvaginal color Doppler US image shows uterine serosa-bladder interface hypervascularity. (C) Sagittal T2-weighted MRI reveal multiple T2-dark bands (arrow) and marked intraplacental vascularity in the lower uterine segment (arrowhead). The patient was clinically diagnosed with PAS. US, ultrasonography; MRI, magnetic resonance imaging; PAS, placenta accreta spectrum. (TIF) [file pone.0349503.s002.tif]

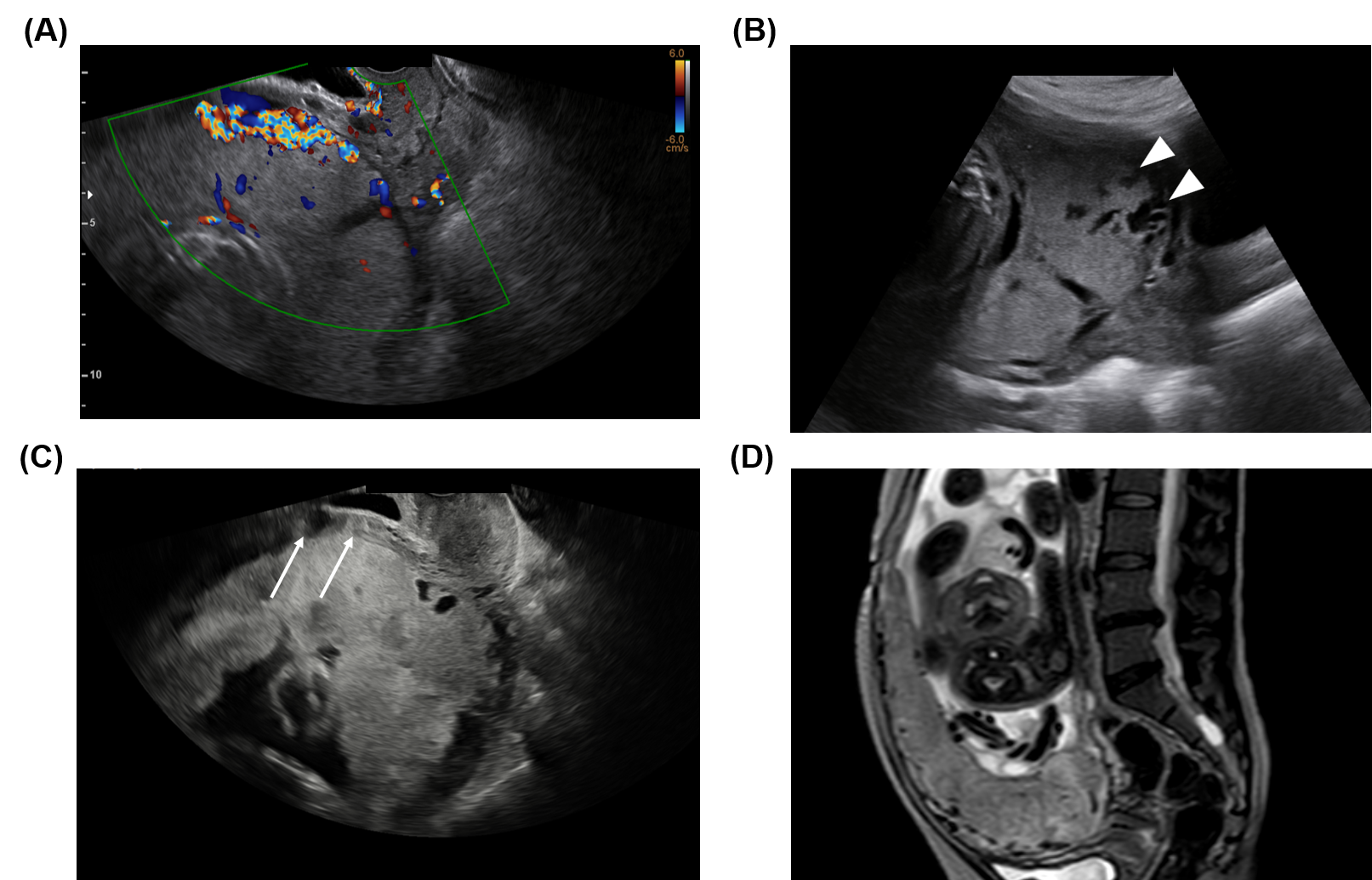

Supplement: S2 Fig — Discordant imaging findings (US-positive/MRI-negative) case in a normal placenta. A 29-year-old woman presented with three US findings highly suggestive of PAS; however, were not corroborated by MRI, and the final diagnosis confirmed a normal placenta (A) Transvaginal color Doppler US showing subplacental hypervascularity. (B) Transabdominal gray-scale US shows multiple lacunae (arrowhead). (C) Transvaginal gray-scale US shows loss of clear zone (arrow). (D) Sagittal T2-weighted MRI shows no imaging features suggestive of placenta accreta. US, ultrasonography; MRI, magnetic resonance imaging. (TIF) [file pone.0349503.s004.tif]

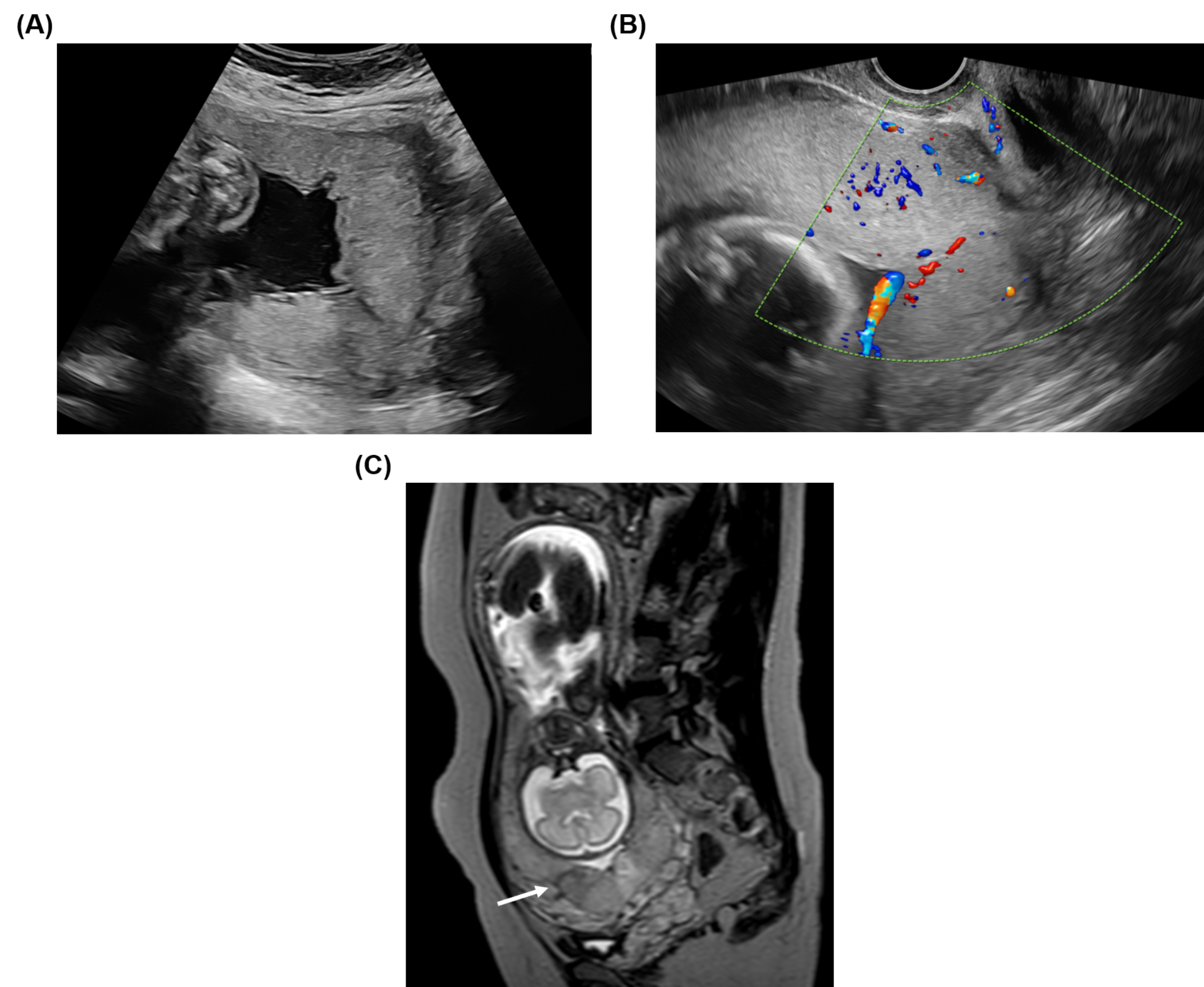

Supplement: S3 Fig — Discordant imaging findings (US-negative/MRI-positive) case in a normal placenta. (A) Transabdominal gray-scale US showing no placental lacunae. (B) Transvaginal color Doppler US shows no signs of subplacental hypervascularity. (C) Sagittal T2-weighted MRI shows multiple T2-dark bands (arrow) suggestive of placenta accreta. US, ultrasonography; MRI, magnetic resonance imaging. (TIF) [file pone.0349503.s006.tif]
