# Supplementary material for: Detection of placenta accreta spectrum and prediction of adverse perinatal outcomes in pregnant women with placenta previa using ultrasonography and magnetic resonance imaging: A retrospective cohort study
Source: PLoS One. 2026 May 29;21(5):e0349503. doi: 10.1371/journal.pone.0349503 (PMC13221029; doi:10.1371/journal.pone.0349503)
Supplement: S2 Table — Perinatal outcomes based on US findings in patients with diagnosed PAS. (DOCX) [file pone.0349503.s003.docx]

**S2 Table.** Perinatal outcomes based on US findings in patients with diagnosed PAS

|  | **PAS-unsuspected on US (*n* = 7)** | **PAS-suspected**  **on US (*n* = 34)** | ***p*-value** |
| --- | --- | --- | --- |
| **Maternal outcomes** |  |  |  |
| EBL (mL) | 1114.3 ± 418.0 | 1733.5 ± 1012.1 | 0.015 |
| Pre-post Hb difference | 3.5 ± 1.5 | 3.0 ± 1.4 | 0.366 |
| Transfusion requirement | 1 (14.3) | 22 (64.7) | 0.031 |
| Transfusion (packs)^*^ | 0.7 ± 1.3 | 2.9 ± 3.7 | 0.139 |
| Intrauterine balloon tamponade | 4 (57.1) | 17 (50.0) | 1.000 |
| Uterine artery embolization | 1 (14.3) | 5 (14.7) | 1.000 |
| Hysterectomy | 0 (0.0) | 13 (38.2) | 0.077 |
| ICU admission | 0 (0.0) | 5 (14.7) | 0.567 |
| **Neonatal outcomes** |  |  |  |
| Preterm birth |  |  |  |
| GA < 37 weeks | 2 (28.6) | 19 (55.9) | 0.238 |
| Birth weight (g) | 3072.9 ± 440.8 | 2728.2 ± 559.3 | 0.134 |
| Birthweight < 2,500 g | 1 (14.3) | 11 (32.4) | 0.651 |
| SGA | 1 (14.3) | 6 (17.6) | 1.000 |
| NICU admission | 2 (28.6) | 14 (41.2) | 0.685 |
| Ventilatory support (intubation) | 0 (0.0) | 10 (29.4) | 0.164 |
| 1-minute AS < 7 | 4 (57.1) | 27 (79.4) | 0.332 |
| 5-minute AS < 7 | 1 (14.3) | 8 (23.5) | 1.000 |

Data are presented as mean ± standard deviation or number (percentage).

^*^Number of packed red blood cell units transfused.

Hb, hemoglobin; AS, Apgar score; GA, gestational age; SGA, small for gestational age; ICU, intensive care unit; EBL, estimated blood loss; NICU, neonatal intensive care unit; PAS, placenta accreta spectrum; SD, standard deviation; US, ultrasonography.
