# Supplementary material for: Detection of placenta accreta spectrum and prediction of adverse perinatal outcomes in pregnant women with placenta previa using ultrasonography and magnetic resonance imaging: A retrospective cohort study
Source: PLoS One. 2026 May 29;21(5):e0349503. doi: 10.1371/journal.pone.0349503 (PMC13221029; doi:10.1371/journal.pone.0349503)
Supplement: S3 Table — Perinatal outcomes based on MRI findings in patients without diagnosed PAS. (DOCX) [file pone.0349503.s005.docx]

**S3 Table.** Perinatal outcomes based on MRI findings in patients without diagnosed PAS

|  | **PAS-unsuspected on MRI (*n* = 82)** | **PAS-suspected**  **on MRI (*n* = 27)** | ***p*-value** |
| --- | --- | --- | --- |
| **Maternal outcomes** |  |  |  |
| EBL (mL) | 1004.3 ± 539.4 | 818.5 ± 478.0 | 0.114 |
| Pre-post Hb difference | 2.5 ± 1.2 | 2.2 ± 1.0 | 0.207 |
| Transfusion requirement | 31 (37.8) | 10 (37.0) | 0.943 |
| Transfusion (packs)^*^ | 0.9 ± 1.3 | 1.1 ± 2.3 | 0.557 |
| Intrauterine balloon tamponade | 44 (53.7) | 17 (63.0) | 0.398 |
| Uterine artery embolization | 5 (6.1) | 2 (7.4) | 0.810 |
| Hysterectomy | 0 (0.0) | 1 (3.7) | 0.080 |
| ICU admission | 0 (0.0) | 1 (3.7) | 0.080 |
| **Neonatal outcomes** |  |  |  |
| Preterm birth |  |  |  |
| GA < 37 weeks | 21 (25.6) | 9 (33.3) | 0.436 |
| Birth weight (g) | 2921.2 ± 356.3 | 2881.1 ± 355.7 | 0.613 |
| Birthweight < 2,500 g | 12 (14.6) | 3 (11.1) | 0.645 |
| SGA | 7 (8.5) | 2 (7.4) | 0.853 |
| NICU admission | 15 (18.3) | 9 (33.3) | 0.102 |
| Ventilatory support (intubation) | 7 (8.5) | 6 (22.2) | 0.057 |
| 1-minute AS < 7 | 42 (51.2) | 18 (66.7) | 0.162 |
| 5-minute AS < 7 | 2 (2.4) | 2 (7.4) | 0.234 |

Data are presented as mean ± standard deviation or number (percentage).

^*^Number of packed red blood cell units transfused.

Hb, hemoglobin; AS, Apgar score; GA, gestational age; SGA, small for gestational age; ICU, intensive care unit; EBL, estimated blood loss; NICU, neonatal intensive care unit; PAS, placenta accreta spectrum; MRI, magnetic resonance imaging.
