# Supplementary material for: Detection of placenta accreta spectrum and prediction of adverse perinatal outcomes in pregnant women with placenta previa using ultrasonography and magnetic resonance imaging: A retrospective cohort study
Source: PLoS One. 2026 May 29;21(5):e0349503. doi: 10.1371/journal.pone.0349503 (PMC13221029; doi:10.1371/journal.pone.0349503)
Supplement: S4 Table — Perinatal outcomes based on MRI findings in patients with diagnosed PAS. (DOCX) [file pone.0349503.s007.docx]

**S4 Table.** Perinatal outcomes based on MRI findings in patients with diagnosed PAS

|  | **PAS-unsuspected on MRI (*n* = 11)** | **PAS-suspected**  **on MRI (*n* = 30)** | ***p*-value** |
| --- | --- | --- | --- |
| **Maternal outcomes** |  |  |  |
| EBL (mL) | 1330.9 ± 631.0 | 1736.7 ± 1046.4 | 0.236 |
| Pre-post Hb difference | 3.1 ± 1.2 | 3.0 ± 1.5 | 0.898 |
| Transfusion requirement | 5 (45.5) | 18 (60.0) | 0.406 |
| Transfusion (packs)^*^ | 0.6 ± 0.8 | 3.2 ± 3.8 | 0.002 |
| Intrauterine balloon tamponade | 5 (45.5) | 16 (53.3) | 0.655 |
| Uterine artery embolization | 0 (0.0) | 6 (20.0) | 0.108 |
| Hysterectomy | 1 (9.1) | 12 (40.0) | 0.060 |
| ICU admission | 0 (0.0) | 5 (16.7) | 0.148 |
| **Neonatal outcomes** |  |  |  |
| Preterm birth |  |  |  |
| GA < 37 weeks | 2 (18.2) | 19 (63.3) | 0.010 |
| Birth weight (g) | 2990.9 ± 454.7 | 2712.3 ± 571.9 | 0.077 |
| Birthweight < 2,500 g | 1 (9.1) | 11 (36.7) | 0.086 |
| SGA | 1 (9.1) | 6 (20.0) | 0.411 |
| NICU admission | 4 (36.4) | 12 (40.0) | 0.833 |
| Ventilatory support (intubation) | 1 (9.1) | 9 (30.0) | 0.167 |
| 1-minute AS < 7 | 7 (63.6) | 24 (80.0) | 0.280 |
| 5-minute AS < 7 | 2 (18.2) | 7 (23.3) | 0.724 |

Data are presented as mean ± standard deviation or number (percentage).

^*^Number of packed red blood cell units transfused.

Hb, hemoglobin; AS, Apgar score; GA, gestational age; SGA, small for gestational age; ICU, intensive care unit; EBL, estimated blood loss; NICU, neonatal intensive care unit; PAS, placenta accreta spectrum; MRI, magnetic resonance imaging.
